# Conventional weight loss therapy in morbid obesity during COVID-19 pandemic: degree of burdens at baseline and treatment efficacy

**DOI:** 10.3389/fpsyt.2024.1330278

**Published:** 2024-01-22

**Authors:** Jessica Schraml, Kerstin Bauer, Sandra Schild, Bea Klos, Rebecca Erschens, Andreas Stengel, Andreas Nieß, Stephan Zipfel, Isabelle Mack

**Affiliations:** ^1^Department of Psychosomatic Medicine and Psychotherapy, University Hospital Tübingen, Tübingen, Germany; ^2^Department for Psychosomatic Medicine, Charité Center for Internal Medicine and Dermatology, Charité-Universitätsmedizin Berlin, Corporate Member of Freie Universität Berlin, Humboldt-Universität zu Berlin and Berlin Institute of Health, Berlin, Germany; ^3^German Center for Mental Health (DZPG), Tübingen, Germany; ^4^Department of Sports Medicine, University Hospital Tübingen, Tübingen, Germany

**Keywords:** Behavioral weight loss, COVID-19, morbid obesity, psychological effects, videoconference-based intervention

## Abstract

**Introduction:**

COVID-19 affected global physical and psychological health. The purpose of this study was to explore the pandemics impact on health-related quality of life (HRQoL), mental health (anxiety, depression, and perceived stress) and eating behavior in people with severe obesity participating in a multimodal conservative behavioral weight loss (BWL) program conducted via videoconferencing. Additionally, the efficacy of the six-month BWL program in a virtual video-based setting during the pandemic was examined.

**Methods:**

297 participants of a face-to-face multimodal behavioral weight loss program prior to the pandemic (PrePAN, May 2014–September 2019) and 146 participants of the in terms of content same intervention in a videoconference-based setting during the pandemic (PAN, July 2020–April 2022) were questioned and compared using standardized questionnaires for HRQoL, symptoms of depressive and anxiety disorders, perceived stress, and eating behavior at baseline and at the end of treatment.

**Results:**

Symptoms for anxiety, depression and perceived stress were similar between PrePAN and PAN at baseline. In addition, PAN tended to show lower disinhibition of eating behavior and feelings of hunger than PrePAN. During the pandemic, the BWL intervention resulted in body weight loss (67%) or stabilization (16%) in most of the participants. It also contributed by improving physical HRQoL, lower worries, and improved eating behaviors compared to baseline.

**Conclusion:**

During the COVID-19 pandemic, baseline mental health of people with morbid obesity was not worse than before the pandemic. Additionally, the BWL intervention in the virtual video-based setting stabilized and improved physical and mental health during the COVID-19 pandemic.

## 1 Introduction

In December 2019, SARS-CoV-2 (Severe acute respiratory syndrome coronavirus type 2) broke out in the Chinese city of Wuhan and spread around the world within weeks. Almost three years after the coronavirus disease-19 (COVID-19) caused by SARS-CoV-2 was declared a global pandemic by the World Health Organization (WHO) (1), nearly all measures to combat the pandemic were dropped in Germany. Between these three years (March 2020 to March 2023), to contain the spread of the virus and thus combat the pandemic, various measures and decrees were enacted of the German government. All involved contact restrictions and severe adjustments to everyday life. Examples include the prohibition of public events as well as bans on the practice of team sports and closure of all sports facilities, new situations in the workplace, and even complete closures of kindergartens and schools (2). Despite the more or less dramatic measures taken by the authorities of the different countries, more than 767 million people were infected by SARS-CoV-2 and nearly seven million deaths were dated due to SARS-CoV-2 worldwide in August 2023 (3).

A global health challenge existing significantly longer than the COVID-19 pandemic is obesity, a pathological increase in body fat mass that is associated with many comorbidities and health risk (4). To prevent a further increase and reduce the current obesity prevalence, prevention and treatment strategies are of great importance. Obesity treatment is mainly achieved through conservative weight-management programs, pharmacotherapy and/or bariatric surgery. Conservative programs are generally multimodal and consist of the fields nutrition, behaviour, and physical activity (5).

Both, obesity and the COVID-19 pandemic are/were associated with stress, impaired HRQoL symptoms of depression, anxiety, and poorly modified eating behaviours (6–17). When both exposures met, severe mutual reinforcement occurred (18–22). For example, obesity was identified as a risk factor for a severe COVID-19 course (23–27) but it appeared to be known only among professionals (28). Infected people with obesity had a 39% increased likelihood of hospitalization (23, 29), increased stays at the Intensive Care Unit (25, 30), higher ventilation rates (26), and an increased mortality (24, 27, 30) as a result of COVID-19 infection. However, not only obesity represented a risk factor for a severe COVID-19 course, but the SARS-CoV-2 outbreak also appeared to be associated with an exacerbation of the obesity epidemic. In particular, an increase in weight during the COVID-19 pandemic was especially found in those individuals who were overweight (BMI 25.0–29.9 kg/m^2^) or obese (BMI ≥ 30.0 kg/m^2^) (18–21). For example, according to the German Health Update (GEDA 2021), more than one in four people living in Germany reported weight gain since the beginning of the corona pandemic (31). People with a higher BMI were more likely to gain weight than those with a BMI within the normal weight range (31). The same results were provided by other European studies and the US (18–20). Conversely, telemedicine dietary intervention during a pandemic could mitigate the negative changes in dietary habits and physical activity that lead to weight gain and thus prevent weight gain during a lockdown (32).

The COVID-19 pandemic has continued to drive technological advances in health care (33). This has enabled the care of patients over a geographical distance, known as “eHealth” or “telemedicine.” Telemedicine online weight loss interventions can be similarly effective as face-to-face BWL programs (34–38), also in older adults in rural areas (39) and as well as during the COVID-19 pandemic (40–43). Furthermore, telemedical interventions can lead to a better physical and psychological wellbeing of people suffering from obesity (44–46).

The purpose of this exploratory study was to examine the impact of the COVID-19 pandemic on mental health and eating behaviours among people with severe obesity attending an online multimodal conservative BWL program.

We hypothesised that (i) individuals with severe obesity were psychologically more distressed and had worse eating behaviour during the COVID-19 pandemic than prior to the pandemic at baseline; (ii) face-to-face BWL intervention during the COVID-19 pandemic lead to physical stabilization and improvement in eating behaviour and psychological parameters.

## 2 Materials and methods

### 2.1 Study design and participants

For this explorative study participants of the VIADUKT program, a multimodal BWL treatment at the University Hospital in Tübingen, Germany, were investigated. The study was approved by the Ethics Committee of the University Hospital Tübingen, Germany with the number 391/2019BO2.

German speaking adults living with obesity were recruited via the multidisciplinary obesity service of the University Hospital in Tübingen and through local general practitioners or specialists. Furthermore, leaflets were distributed among practitioners in and around Tübingen, and the website of the University Hospital was used for recruitment.

To uncover differences in baseline characterization of participants prior to and during the COVID-19 pandemic (i), the baseline characteristics of two groups within VIADUKT were investigated. Group 1, named “PrePAN,” consists of 297 subjects who participated in the VIADUKT program prior to the COVID-19 pandemic (from May 2014 until September 2019, face-to-face), being characterized previously (47). Group 2, “PAN” includes 146 subjects which participated in the program during the COVID-19 pandemic (from July 2020 until April 2022, virtual, video-based). To create a clear distinction between the pandemic period and the pre-pandemic period, participants from courses that started prior to the COVID-19 pandemic but were completed during the pandemic were excluded from the analyses.

To test the effectiveness of the BWL program during the COVID-19 pandemic (ii), all participants of PAN were analysed regarding subjectively measured body weight, body height, HRQoL, anxiety, depression, distress, and eating behaviour between pre- (T0, two weeks prior to the intervention) and posttreatment (T1, one week before the intervention ended). Due to less stringent lockdown policies in the summer months of 2020 and 2021, single units may have been conducted face-to-face and not online. As we did not find any differences between courses with at least 80% online sessions and online-only courses, all these online courses were included in this analysis. Furthermore, a subgroup analysis was performed according to the attitude towards bariatric surgery since this is an important factor for conservative treatment success (47). This attitude was recorded in the questionnaire at the beginning of the intervention. Participants who considering bariatric surgery at T0 were assigned to PAN_POS_ (*n* = 90). Those who excluded bariatric surgery at T0 were named PAN_NEG_ (*n* = 53). Participants with an uncertain attitude (PAN_Uncert_, *n* = 3) were not included in subgroup analyses.

Due to the heterogeneous conditions between online and face-to-face courses (online courses during COVID-19 pandemic, face-to-face courses before COVID-19 pandemic), no direct comparisons were made between PrePAN and PAN.

### 2.2 Treatment (VIADUKT)

“VIADUKT” is a multimodal conventional BWL program for patients with predominantly severe obesity (grade III, mean BMI = 42.7 kg/m^2^). The program includes theory sessions as well as practical exercise sessions conducted by a multidisciplinary team consisting of nutritional, psychological and sports medicine specialists. The theory sessions focus on nutritional education and behavioural therapy to promote lifestyle change. Specifically, participants are trained on motivational enhancement, flexible but controlled eating behaviours, basics of physical activity, stress management techniques, and strategies for permanent weight loss. The program meets the requirements of the German guideline for the clinical treatment of obesity (48).

The intervention takes place at two-week intervals over a period of six months. Due to the COVID-19 pandemic, the group sessions originally held face-to-face at the hospital were moved to a synchronous virtual, video-based privacy-compliant online platform. Prior to the pandemic, twelve participants attended ten 75-min educational group sessions and twenty 45-min guided exercise sessions. As a result of the pandemic-related shift to video-based training, these ten educational theory sessions were reduced to 60 min per session but not including not more than six participants. Additional, material and homework were provided one week ahead of each theory online class via e-mail. Practical sessions were transformed to ten 60-min sessions. If participants attended at least 80% of the group sessions and physical activity meetings, the program was considered complete, otherwise a dropout was declared.

### 2.3 Outcomes

For PrePAN, the outcomes are reported in details elsewhere (47). Except of body weight assessment, measurements for the PrePAN and the PAN sample were identical. Body weight of the PrePAN individuals was assessed at site, using a calibrated scale, wearing only light clothes and no shoes. Body height and body weight of the PAN-individuals was self-reported. The participants used their own body scales at home. Although subjectively measured body weight is never as accurate as objectively measured body weight, especially in people living with obesity (49), there are also studies showing that self-reported body weight is precise, even in people living with obesity (50) and especially for people living with obesity who participate a weight loss program (51).

Body weight change in kilograms over the six-month intervention period was calculated for PAN. As reported before, all body weight and height values are self-reported by the participants. The BMI of the participants was calculated from the information of height and subjectively measured body weight.

Validated questionnaires were used for HRQoL (SF-12), anxiety (GAD-7), depression (PHQ-9), distress (PSQ20), and eating behaviour (TFEQ) at T0 and T1. The applied questionnaires are described in detail below. For the subgroup analyses, the participant’s attitudes towards bariatric surgery were assessed with an open question called “What is your attitude towards bariatric surgery?.” The answers were categorized into: “Yes, bariatric surgery is an option for me” (PAN_POS_), “No, bariatric surgery is not an option for me” (PAN_NEG_), “I am not sure” (PAN_Uncert_). The results are presented for the total PAN group and for the two subgroups PAN_POS_ versus PAN_NEG_, but not for the PAN_Uncert_.

The *Short Form Health-Survey 12 (SF-12)* is used to assess HRQoL, consisting of 12 items of mental and physical component summaries. The average score is 50 (SD = 10) for each. Higher scores represent better health status, whereas lower scores represent poorer referred to the general U.S. population (52).

For screening generalized anxiety disorder, the *Generalized Anxiety Disorder Scale (GAD-7)* is used. Patients rate the 7 items of GAD-7 with the options “not at all,” “on some days,” “on more than half of the days” and “almost every day.” Scores range between 0–21 and were categorized into minimal (0–4), mild (5-9), moderate (10-14) or severe (15-21) anxiety (53).

The occurrence and severity of depressive disorder is assessed using the 9-item *Patient Health Questionnaire (PHQ-9)*. Possible answer options are “not at all,” “several days,” “more than half of the days” or “nearly every day.” The calculated score is categorized into none to minimal (0–4), mild (5-9), moderate (10-14), moderately severe (15-19) or severe (20-27) levels of depression (54).

Perceived stress is measured by the German version of the *Perceived Stress Questionnaire-20 (PSQ20)*. It consists of 20 items, which are divided into four subscales and represent the distress-determining constructs “worry,” “tension,” “pleasure,” and “demands.” In addition, a sum score is calculated. All statements are to be answered on a 4-point-Likert-scale ranging from “almost never,” “sometimes,” “frequently” to “most of the time.” The result is a scale rank between 0 and 100 with high values on a subscale meant to be a high expression of the respective construct (55).

The German version of the *Three-Factor Eating Questionnaire (TFEQ),* which contains 51 items of 3 subscales “Cognitive Restraint,” “Disinhibition,” and “Feelings of Hunger,” efforts on eating behaviour. High scores at the subscales represent high expressions of the constructs (56).

These questionnaires have been used and validated in patients with obesity [HRQoL (57, 58), anxiety and depression (59, 60), perceived stress (55, 61) and eating behaviour (62–64)].

Additionally, at the end of the intervention the participants completed an evaluation questionnaire that captured participant satisfaction with the program in 10 questions regarding *personal benefits, importance and usefulness of the sessions, adequacy of the exercise level, motivation to implement more exercise into daily life, whether participants felt prepared for the time after the program and optimism to maintain or further reduce the weight*. Participants responded to the questions using a 5-point Likert scale from “strongly disagree” (1),” disagree” (2), “neither agree nor disagree” (3), “agree” (4) to “strongly agree” (5). Additionally, participants were asked if they would recommend the program to family and friends.

### 2.4 Statistical analysis

Statistical analysis was performed using IBM SPSS Statisics for Windows software, version 28.0 (65). Data are reported as mean (standard deviation, SD), confidence interval (CI) along with median with 25th and 75th percentiles (interquartile range, IQR). Frequencies are expressed as percentages (%). Normal distribution was tested using Kolmogorov–Smirnov tests, and equality of variances between groups was tested using Levene’s tests.

To explore the first hypothesis (i), differences in baseline-characterization between the two groups of the total sample (PrePAN and PAN) were detected with metric data and simultaneous normal distribution using the *t*-test. Non-normally distributed data were determined with the Mann–Whitney U test. For nominal data, the χ^2^-test or, if the expected frequencies of the cells were too low, the Fisher–Freeman–Halton’s exact test was used. In addition, to detect possible differences due to unequal group sizes, cases of PrePAN were matched with cases of PAN according to age, sex, BMI, and attitude toward bariatric surgery (hereafter referred to as PrePAN_Match_ and PAN_Match_).

To test for the second hypothesis (ii), differences in self-reported body weight, HRQoL, anxiety, depression, perceived stress, and eating behaviors between beginning of the intervention (T0) and the end (T1) for the whole PAN group (PAN_NEG_, PAN_POS_ and PAN_Uncert_) were analysed using the Wilcoxon signed-rank test. To analyse subgroup (PAN_NEG_ vs. PAN_POS_) and time (T0 vs. T1) interactions in body weight, BMI, and psychometrics, a 2×2 ANOVA was performed with main effects for time as a within-subjects variable and subgroup as a between-subjects variable. Body weight reductions of ≥5% of baseline weight are considered clinically significant body weight reductions (5, 66). Body weight reductions up to 4.9% of baseline weight are considered low, from 5.0%–9.9% are considered moderate, and >10% are considered high body weight reductions (47). Weight gain <2 kg are considered weight stabilization (20, 67). Two-tailed tests are used throughout the outcome calculation.

To control for α-error, *p*-values of psychological outcomes were adjusted for the *false discovery rate (FDR)* in multiple testing (68). FDR values <0.001 were considered statistically highly significant. FDR values between 0.001 and 0.05 were considered statistically significant. FDR values between 0.05 and 0.1 were assessed as a trend. For age, baseline body weight, and baseline BMI, as well as for demographics at baseline and for the body weight change, *p*-values were reported because they were not affected by multiple testing. *p*-values <0.001 were considered statistically significant. *p*-values between 0.001 and 0.05 were considered a trend. Effect sizes for the χ2 -test and Mann–Whitney U test are defined as follows: r-(φ-) of 0.1 = low effect, 0.3 = medium effect, 0.5 = high effect (69).

### 2.5 Missing data imputation

Missing Data were replaced by the predictive mean-matching method after using Little’s test to determine whether missing data were random (70).

For the per protocol population, participants were only included if body weight (kg) was provided at T0 and T1 of intervention, and if they were not classified as dropout (≥80% exposure). After excluding the participants with missing data at T0 (*n* = 8) and/or the non-completer (*n* = 13), the per protocol population consisted of 132 participants,

The intention-to-treat population consisted of 146 participants for the body weight change and 138 for the psychological parameters (due to *n* = 8 for missing questionnaire data at T0). Age, sex, and self-reported body weight at baseline were chosen as predictors for the body weight change. For the psychological parameters, age and sex served as predictors. To fill in gaps in the assessment of HRQoL, depressive and anxiety disorders, stress, and eating behaviours, multiple imputation with five iterations was performed both for single missing values (at intervention beginning and end) and for completely missing questionnaires (at the end of intervention only) (71, 72). Cases with complete missing questionnaires and/or single-item at the end imputations were included in the intention-to-treat (ITT) population. The data were analysed for the per protocol population and for the full-data analysis. Since no differences between the approaches were found, the results of the full-data analysis are presented.

The percentage of missing data for complete questionnaires ranged from 5% to 30% [T0 HRQoL = 6.2% (*n* = 10), T1 HRQoL = 30.1% (*n* = 44), T0 anxiety = 7.5% (*n* = 11), T1 anxiety = 30.1% (*n* = 44), T0 depression = 7.5% (*n* = 11), T1 depression = 30.1% (*n* = 44), T0 eating behaviour = 5.5% (*n* = 8), T1 eating behaviour = 30.1% (*n* = 44), T0 stress = 5.5% (*n* = 8), T1 stress = 30.1% (*n* = 44)].

### 2.6 Further analyses

Minimal clinically important differences (MCIDs) were reported when possible. For anxiety and depression, MCIDs were set to a value of four points (53, 72). For HRQoL, a score of three points was assumed to be the minimum clinically relevant change (71). Furthermore, scores for generalized anxiety disorder and for depressive disorder as well as for eating behaviours were divided into subgroups according to the manuals (depression: 0–4 minimal, 5–9 mild, 10–14 moderate, 15–21 severe depression; anxiety: 0–4 minimal, 5–9 mild, 10–14 moderate, 15–21 severe anxiety; TFEQ subscale 1: 0–3 very low, 4–6 low, 7–9 moderate, 10–13 high, 14–21 very high Cognitive Restraint; TFEQ subscale 2: 0–3 very low, 4–5 low, 6–8 moderate, 9–11 high, 11–16 very high Disinhibition; TFEQ subscale 3: 0–2 very low, 3–4 low, 5–6 moderate, 7–9 high, 10–14 very high Feelings of hunger). The percentages of participants who attained MCIDs or achieved a change in class on TFEQ (improvement or worsening) or remained the same in their class are reported.

## 3 Results

### 3.1 Group characteristics

A total of 443 participants with predominantly (65%) grade III obesity who participated in the VIADUKT program since May 2014 until April 2022 were included. Most participants (77%) were women with a mean age of 42.2 years (SD = 12.4) and a mean BMI of 42.7 kg/m^2^ (SD = 5.4). In the following, the participants of the total sample are divided into two groups, PrePAN and PAN.

The PrePAN group has been described in detail elsewhere (47). In short, prior to the COVID-19 pandemic, 297 probands participated in the face-to-face VIADUKT program (PrePAN). Almost all (92%) participants completed the intervention. The drop-out rate was 8%. The group consisted predominantly of women (76%) and two third of the patients had grade III obesity (65%). 38% had a negative attitude towards bariatric surgery (PrePAN_NEG_), whereas 37% had a positive (PrePAN_POS_). 10% were undecided (PrePAN_Uncert_) and 15% did not provide any information on their attitude.

The PAN group consists of 146 subjects who participated in the VIADUKT program in a virtual, video-based setting during the COVID-19 pandemic, from July 2020 until April 2022. Out of these, 12 subjects attended less than 80% of the meetings, so the dropout rate was 8%. The majority (80%) of the participants were women and had obesity grade III (63%). More than half (62%) of the participants declared to not desire bariatric surgery (PAN_NEG_), whereas 53 (36%) participants wanted to receive bariatric surgery (PAN_POS_). Only three (2%) participants were undecided (PAN_Uncert_).

### 3.2 People with severe obesity were psychologically not more distressed or had worse eating behaviour during the COVID-19 pandemic than prior to the pandemic at baseline

PrePAN and PAN did not differ significantly from each other with respect to age, initial weight, and BMI as well as in sociodemographic data (*p* > 0.05). Furthermore, there was no significant difference in HRQoL (FDR >0.1). In contrast, PAN showed significantly lower scores for anxiety and depression, as well as stress, disinhibition of eating behaviour and feelings of hunger (FDR <0.05). A detailed overview of the baseline characteristics for PrePAN and PAN is presented in Table 1. Since the PrePAN group was almost twice as large as the PAN group, matched population analyses were also performed. Detailed baseline characterization for the two sample groups matched by age, sex, initial BMI, and attitude towards bariatric surgery are shown in Supplementary material S1. PrePAN_Match_ and PAN_Match_ did not differ significantly in sociodemographic data (*p* > 0.05). In contrast to the full population analysis (PrePAN versus PAN), there were no differences in HRQoL, anxiety, depression, and perceived stress between PrePAN_Match_ and PAN_Match_ (FDR >0.1). However, in line with PrePAN and PAN, in the matched population PAN_Match_ participants tended to have lower scores in the subscales Disinhibition and Feelings of Hunger of the TFEQ.

**Table 1:** Baseline characterization of the total group (*N* = 443).

	prePAN (*n* = 297)	PAN (*n* = 146)	Statistics for prePAN vs. PAN
	mean (SD) [95% CI]	mean (SD) [95% CI]	Mann–Whitney U Test/χ2
Age [years]	41.5 (12.1) [40.1–42.9]	42.3 (13.0) [40.2–44.4]	*U* = 21011.000, *p* = 0.597
BMI [kg/m^2^]	42.7 (5.4) [42.1–43.4]	42.6 (5.6) [41.7–43.6]	*U* = 20814.000, *p* = 0.530
Range: min to max	27.6–63.3	28.9–66.3	
Weight [kg]	123.6 (21.0) [121.2–126.0]	123.2 (19.4) [120.0–126.4]	*U* = 21541.500, *p* = 0.912
Range: min to max	74–184	75.8–185	
	*N* (%)	*N* (%)	
Sex (female)	225 (76)	116 (80)	χ2 (1, *N* = 443) = 0.497, *p* = 0.481, φ = −0.033
Nationality			χ2 (1, *N* = 398) = 0.422, *p* = 0.516, φ = 0.033
German/foreigner	229 (86)/36 (14)	118 (89)/15 (11)	
Smoker	60 (23)	24 (17)	χ2 (1, *N* = 400) = 1.654, *p* = 0.198, φ = −0.064
Bariatric surgery desire			χ2 (3, *N* = 443) = 38.978, **p < 0.001**, φ = 0.297
No	113 (38)	90 (62)	
Yes	111 (37)	53 (36)	
Not clear	30 (10)	3 (2)	
Unknown	43 (14)	0 (0)	
Personal status			χ2_FFH_ (5, *N* = 397) = 3.855, *p* = 0.570, φ = 0.099
Single	83 (32)	35 (25)	
Married	140 (54)	85 (62)	
Separated	5 (2)	2 (1)	
Divorced	21 (8)	13 (9)	
Widowed	4 (2)	2 (1)	
Others	6 (2)	1 (1)	
Composition of household			χ2_FFH_ (6, *N* = 395) = 1.148, *p* = 0.979, φ = 0.054
Alone	40 (15)	19 (14)	
With partner	67 (26)	40 (29)	
Alone with child(ren)	19 (7)	10 (7)	
Partner and child(ren)	93 (36)	49 (36)	
With parents	27 (10)	13 (10)	
Others	13 (5)	5 (4)	
Level of education			χ2_FFH_ (6, *N* = 396) = 10.128, *p* = 0.119, φ = 0.160
Sec. mod. school	78 (30)	39 (29)	
Polytechnic	3 (1)	0 (0)	
Sec. techn. school	87 (34)	51 (37)	
High school	39 (15)	32 (23)	
University	43 (17)	13 (10)	
Others	8 (3)	2 (2)	

	prePAN		PAN		Statistics for prePAN vs. PAN
	Mean (SD) [95% CI]	Median [IQR]	Mean (SD) [95% CI]	Median [IQR]	Mann Whitney U test
HRQoL (SF-12)					
MCS	42.4 (11.4) [41.0–43.7]	42.1 [34.9–51.9]	43.9 (11.9) [41.9–45.9]	43.9 [35.1–55.3]	*U* = 17677.000, *FDR* = 0.251
PCS	35.5 (11.2) [33.9–36.5]	34.3 [26.6–44.6]	33.7 (11.0) [31.9–35.6]	32.5 [24.7–42.7]	*U* = 17796.000, *FDR* = 0.251
Anxiety (GAD-7) score	7.8 (4.8) [7.3–8.4]	7 [5–11]	6.7 (4.6) [5.9–7.5]	6 [3–9]	*U* = 16294.500, *FDR* = 0.029, *r* = −0.12
Depression (PHQ-9) score	9.3 (5.2) [8.7–9.9]	9 [6–12.8]	8.1 (5.0) [7.3–9.0]	7 [4–11]	*U* = 16279.500, *FDR* = 0.029, *r* = −0.12
Eating behavior (TFEQ) scores					
TFEQ-subscale: cognitive restraint	8.5 (3.9) [8.0–9.0]	8 [6–11]	8.1 (4.5) [7.4–8.9]	8 [5–11]	*U* = 17743.000, *FDR* = 0.251,
TFEQ-subscale: disinhibition	9.9 (3.5) [9.4–10.3]	10 [7–13]	8.7 (3.6) [8.1–9.3]	8 [6–11]	*U* = 15587.000, *FDR* = 0.011, *r* = −0.15
TFEQ-subscale: feelings of hunger	7.9 (3.4) [7.5–8.3]	8 [5–10.8]	6.4 (3.6) [5.8–7.0]	6.5 [3–9]	*U* = 14707.000, *FDR* = 0.001, *r* = −0.19
Perceived stress (PSQ20) scores					
Sum	51.4 (20.5) [48.8–53.9]	53.3 [36.7–66.7]	46.7 (20.1) [43.3–50.1]	43.3 [33.3–63.3]	*U =* 15017.500, *FDR* = 0.033, *r* = −0.12
Worries	48.7 (25.6) [45.5–51.8]	53.3 [26.7–66.7]	43.0 (26.4) [38.6–47.5]	40 [26.7–61.7]	*U =* 15294.000, *FDR* = 0.055
Tension	54.5 (23.9) [51.6–57.5]	53.3 [40–73.3]	50.1 (24.1) [46.1–54.2]	46.7 [33.3–66.7]	*U =* 15539.500, *FDR* = 0.083
Joy	46.7 (22.4) [43.9–49.4]	46.7 [26.7–66.7]	48.8 (20.7) [45.3–52.3]	46.7 [33.3–60]	*U =* 16475.000, *FDR* = 0.300
Demands	48.9 (24.5) [45.9–52.0]	46.7 [33.3–66.7]	42.5 (22.9) [38.6–46.1]	40 [26.7–55.0]	*U =* 14704.500, *FDR* = 0.028 *r* = −0.14

BMI, body mass index; CI, confidence interval; FDR, false discovery rate; GAD, generalized anxiety disorder scale; IQR, interquartile range; HRQoL, health-related quality of life; MCS, mental component score; n, sample size; PCS, physical component score; PHQ, patient health questionnaire; PAN, patients participating in the intervention since the COVID-19 pandemic; prePAN, Patients participating in the intervention prior to the COVID-19 pandemic; PSQ, perceived stress questionnaire; SD, standard derivation; SF, short form health survey; TFEQ, three factor eating questionnaire; T0, in the beginning of the intervention; T1, at the end of the intervention. Statistics: U = Mann–Whitney U test; χ2 = χ2 test; φ = effect size (for χ2 test); *p* < 0.001 is considered statistically significant; FDR < 0.05 is considered statistically significant, FDR 0.05–0.1 is considered as a trend, *r* = effect size (for Mann–Whitney U test). Significant changes and differences were marked in bold.

### 3.3 Effectiveness of the intervention during the COVID-19 pandemic

#### 3.3.1 Patients’ physical situation and health related quality of life was stabilized in most participants

A detailed overview of the body weight change data is presented in Supplementary material S2. In the per protocol population the mean percentage reduction of subjectively measured body weight for PAN was 1.5%. This equates to a mean body weight change of −1.9 kg (SD = 5.5), ranging from −25.3 kg to +11.9 kg (*z* = −4.148, *p < 0*.001, *n* = 132, *r* = −0.36). Since the treatment efficacy differs in people with positive versus negative attitude towards bariatric surgery at the beginning of the treatment (47), the results are also reported for these subgroups. PAN_NEG_ refers to the group with a negative attitude towards bariatric surgery and PAN_POS_ to the group with a positive attitude. Mean percentage reduction of subjectively measured body weight was 2.3% for PAN_NEG_ and 0.04% for PAN_POS_, equating to a mean body weight change of −2.8 kg (SD = 4.7) and −0.1 kg (SD = 6.3), respectively (*F*(1,129) = 7.852, *p = 0*.006, partial η^2^ = 0.1). In the intention-to-treat population the mean percentage reduction of subjectively measured body weight was 1.5%, which equates to a mean body weight change of −1.9 kg (SD = 5.2; z = −4.613, *p* < 0.001, *n* = 146, r = −0.38). In comparison, the mean percentage reduction of subjectively measured body weight was 2.2% for the PAN_NEG_ (*n* = 90) and 0.2% for the PAN_POS_ (*n* = 53), equating to a mean body weight change of −2.7 kg (SD = 4.5) and −0.3 kg (SD = 6.1), respectively (*F*(1,143) = 7.496, *p = 0*.007, partial η^2^ = 0.1).

In line, the physical component summary of the HRQoL questionnaire (SF-12) tended to be improved (Table 2) whereas the mental component summary remained stable. For PAN_POS_, the mental component summary improved over the course of time, while PAN_NEG_ showed significant improvements for the physical component summary (Table 3).

**Table 2:** Anthropometrical and psychological outcomes of the whole study population.

PAN	T0 (*N* = 138)		T1 (*N* = 138)		Statistics for T0vs. T1
	Mean (SD) [95% CI]	Median [IQR]	Mean (SD) [95% CI]	Median [IQR]	Wilcoxon
BMI [kg/m^2^]	42.6 (5.6) [41.7–43.6]	42.1 [38.8–45.7]	42.0 (5.9) [41.1–43.0]	41.1 [37.8–45.7]	*z =* −4.482, *p* < *0*.001 *r =* −0.37
Weight [kg]	123.2 (19.4) [120.0–126.4]	120.6 [110.0–136.0]	121.3 (19.5) [118.1–124.5]	118.8 [107.3–135.2]	*z =* −4.613, *p* < *0*.001 *r =* −0.38
HRQoL					
MCS	43.9 (11.9) [41.9–45.9]	43.9 [35.1–55.3]	44.1 (12.0) [42.1–46.1]	45.7 [32.7–54.4]	*z =* −0.325, *FDR =* 0.745,
PCS	33.7 (11.0) [31.9–35.6]	32.5 [24.7–42.7]	36.0 (11.9) [34.0–38.0]	37.7 [25.9–45.9]	*z =* −2.044, *FDR =* 0.070, *r* = −0.17
Anxiety (GAD-7) score	6.7 (4.6) [5.9–7.5]	6 [3–9]	6.3 (4.4) [5.6–7.1]	6 [3–8.3]	*z =* −0.570, *FDR =* 0.620
Depression (PHQ-9) score	8.1 (5.0) [7.3–9.0]	7 [4–11]	7.4 (4.5) [6.7–8.2]	7 [4–10]	*z =* −1.627, *FDR =* 0.156
Eating behavior (TFEQ) scores						
TFEQ-subscale: cognitive restraint	8.1 (4.5) [7.4–8.9]	8 [5–11]	10.9 (4.0) [10.2–11.5]	11 [8–14]	*z =* −6.297, *FDR <* **0.001,** *r =* 0.54
TFEQ-subscale: disinhibition	8.7 (3.6) [8.1–9.3]	8 [6–11]	6.0 (2.9) [5.5–6.5]	6 [4–8]	*z =* −7.873, *FDR <* **0.001,** *r =* 0.67
TFEQ-subscale: feelings of hunger	6.4 (3.6) [5.8–7.0]	6.5 [3–9]	4.7 (3.2) [4.1–5.2]	4 [2–7]	*z =* −5.249, *FDR <* **0.001,** *r =* 0.45
Perceived stress (PSQ20) scores						
Sum	46.7 (20.1) [43.3–50.1]	43.3 [33.3–63.3]	42.5 (17.6) [39.6–45.5]	41.7 [30.0–51.7]	*z =* −2.318, *FDR =* 0.041, *r =* −0.20
Worries	43.0 (26.4) [38.6–47.5]	40.0 [26.7–61.7]	36.1 (21.6) [32.5–39.8]	33.3 [20.0–46.7]	*z =* −3.541, *FDR =* 0.001 *r =* −0.30
Tension	50.1 (24.1) [46.1–54.2]	46.7 [33.3–66.7]	44.7 (21.1) [41.1–48.2]	40.0 [33.3–55.0]	*z =* −2.567, *FDR =* 0.024 *r =* −0.22
Joy	48.8 (20.7) [45.3–52.3]	46.7 [33.3–60.0]	51.7 (19.8) [48.4–55.1]	53.3 [40.0–60.0]	*z =* −1.435, *FDR* = 0.202
Demands	42.5 (22.9) [38.6–46.3]	40.0 [26.7–55.0]	41.0 (20.7) [37.5–44.5]	40.0 [26.7–53.3]	*z =* −0.773, *FDR* = 0.528

BMI, body mass index; CI, confidence interval; FDR, false discovery rate; GAD, generalized anxiety disorder scale; IQR, interquartile range; HRQoL, health-related quality of life; MCS, mental component score; *N*, sample size; PCS, physical component score; PHQ, patient health questionnaire; PAN, patients participating in the intervention during the COVID-19 pandemic; PSQ, perceived stress questionnaire; SD, standard derivation; TFEQ, three factor eating questionnaire; T0, in the beginning of the intervention; T1, at the end of the intervention. Statistics: *p* < 0.001 is considered statistically significant; FDR < 0.001 is considered highly statistically significant; FDR < 0.05 is considered statistically significant, FDR 0.05–0.1 is considered a trend, *r* = effect size, *z* = Wilcoxon test. Significant differences or changes were marked in bold.

**Table 3:** Psychological outcomes for PAN_NEG_ and PAN_POS._

Variable	PAN_NEG_	PAN_POS_	Statistics (2×2 ANOVA)
*n*	84	51	
**Anxiety (GAD-7)**			F (1, 133) = 0.901, *FDR* = 0.689
Mean_Post_ (SD) [95% CI]	6.2 (3.6) [5.5–7.0]	6.7 (5.5) [5.1–8.2]	
ΔMean between T0 and T1 (SD)	−0.6 (4.4)	−0.8 (5.0)	
Median_Post_ [IQR]	6 [4–8.8]	5 [3–10]	
MCID improved (%)	16 (19)	12 (24)	
MCID did not changed (%)	49 (58)	31 (61)	
MCID worsened (%)	19 (23)	8 (16)	
**Depression (PHQ-9)**			F (1, 133) = 2.296, *FDR* = 0.528
Mean_Post_ (SD) [95% CI]	7.2 (4.2) [6.3–8.2]	7.8 (4.9) [6.5–9.3]	
ΔMean between T0 and T1 (SD)	−0.3 (4.7)	−1.5 (5.1)	
Median_Post_ [IQR]	6 [4.3–9]	7 [4–11]	
MCID improved (%)	19 (23)	12 (24)	
MCID did not changed (%)	51 (61)	33 (65)	
MCID worsened (%)	14 (17)	6 (12)	
**HRQoL (SF-12)**			F (1, 133) = 5.619, *FDR* = 0.230 *F* (1, 133) = 2.829, *FDR* = 0.528
**MCS**			
Mean_Post_ (SD) [95% CI]	42.7 (11.7) [40.2–45.2]	45.6 (12.3) [42.2–45.8]	
ΔMean between T0 and T1 (SD)	−1.9 (12.3)	+3.4 (13.2)	
Median_Post_ [IQR]	42.6 [32.3–53.2]	49.4 [34.0–55.1]	
MCID improved (%)	22 (26)	28 (55)	
MCID did not changed (%)	26 (31)	10 (20)	
MCID worsened (%)	36 (43)	13 (26)	
**PCS**			
Mean_Post_ (SD) [95% CI]	39.3 (10.6) [37.0–41.6]	30.9 (12.3) [27.5–34.4]	
ΔMean between T0 and T1 (SD)	+3.6 (10.1)	+0.6 (10.0)	
Median_Post_ [IQR]	41.5 [32.3–48.7]	28.3 [21.8–39.8]	
MCID improved (%)	42 (50)	14 (28)	
MCID did not changed (%)	22 (26)	12 (24)	
MCID worsened (%)	20 (24)	25 (49)	
**Eating behaviour (TFEQ)**			
**Cognitive restraint**			
Mean_Post_ (SD) [95% CI]	10.9 (3.7) [10.1–11.7]	10.7 (4.4) [9.5–12.0]	F (1, 133) = 0.001, *FDR* = 0.994
ΔMean between T0 and T1 (SD)	+2.7 (5.0)	+2.7 (3.5)	
Median_Post_ [IQR]	11.5 [8.3–14]	11 [7–14]	
**Disinhibition**			
Mean_Post_ (SD) [95% CI]	5.7 (2.9) [5.1–6.4]	6.6 (3.0) [5.7–7.4]	F (1, 133) = 0.000, *FDR* = 0.994
ΔMean between T0 and T1 (SD)	−2.8 (3.6)	−2.7 (3.0)	
Median_Post_ [IQR]	6 [3.3–7]	6 [4–9]	
**Feelings of hunger**			
Mean_Post_ (SD) [95% CI]	4.3 (2.9) [3.7–4.9]	5.4 (3.6) [4.4–6.4]	F (1, 133) = 1.631, *FDR* = 0.556
ΔMean between T0 and T1 (SD)	−2.0 (3.4)	−0.9 (3.0)	
Median_Post_ [IQR]	4 [2–6]	5 [3–9]	
**Perceived stress (PSQ20)**			
**Sum**			
Mean_Post_ (SD) [95% CI]	41.6 (16.9) [38.0–45.3]	44.8 (18.7) [39.6–50.1]	F (1, 133) = 0.150, *FDR* = 0.932
ΔMean between T0 and T1 (SD)	−3.7 (16.6)	−4.8 (17.0)	
Median_Post_ [IQR]	41.7 [30.0–49.6]	43.3 [31.7–56.7]	
**Worries**			
Mean_Post_ (SD) [95% CI]	35.0 (19.0) [30.9–39.1]	39.1 (25.3) [32.0–46.2]	F (1, 133) = 0.586, *FDR* = 0.763
ΔMean between T0 and T1 (SD)	−5.6 (19.8)	−8.5 (24.3)	
Median_Post_ [IQR]	33.3 [21.7–45.0]	33.3 [20.0–53.3]	
**Tension**			
Mean_Post_ (SD) [95% CI]	43.1 (19.4) [38.9–47.3]	48.5 (23.6) [41.9–55.1]	
ΔMean between T0 and T1 (SD)	−5.5 (23.2)	−5.3 (22.2)	
Median_Post_ [IQR]	40.0 [33.3–53.3]	46.7 [33.3–60.0]	
**Joy**			
Mean_Post_ (SD) [95% CI]	51.0 (18.6) [47.0–55.1]	52.3 (22.1) [46.1–58.5]	F (1, 133) = 1.445, *FDR* = 0.556
ΔMean between T0 and T1 (SD)	+1.2 (18.4)	+5.4 (20.9)	
Median_Post_ [IQR]	53.3 [40.0–60.0]	53.3 [33.3–73.3]	
**Demands**			
Mean_Post_ (SD) [95% CI]	39.5 (21.1) [34.9–44.1]	44.1 (19.9) [38.5–49.6]	F (1, 100) = 0.403, *FDR* = 0.790
ΔMean between T0 and T1 (SD)	−2.5 (19.1)	−0.3 (22.0)	
Median_Post_ [IQR]	40.0 [26.7–53.3]	40.0 [33.3–53.3]	

BMI, body mass index; CI, confidence interval; FDR, false discovery rate; GAD, generalized anxiety disorder scale; IQR, interquartile range; HRQoL, health-related quality of life; MCS, mental component score; *n*, sample size; PCS, physical component score; PHQ, patient health questionnaire; PAN_NEG_, patients with a negative attitude towards bariatric surgery participating in the intervention during the COVID-19 pandemic; PAN_POS_, Patients with a positive attitude towards bariatric surgery participating in the intervention prior to the COVID-19 pandemic; PSQ, perceived stress questionnaire; SD, standard derivation; SF, Short form health survey; TFEQ, three factor eating questionnaire; T0, in the beginning of the intervention; T1, at the end of the intervention. Statistics: F = ANOVA; FDR < 0.05 is considered statistically significant.

Categorized according to MCIDs of the PAN group, the physical score improved in 41%, worsened in 34% and remained stable in 25% of the patients; the mental score improved in 37%, worsened in 36% and remained stable in 27% of the participants.

#### 3.3.2 Stress perception and eating behaviour improved while depression and anxiety symptoms remained stable

The sum score for perceived stress improved significantly during the intervention period (Table 2). Additionally, the subscale “worries” and “tension” improved significantly from pre- to post-treatment. PAN_NEG_ showed significant improvements for “worries” subscale, and they tended to show improved scores for subscale “tension” (Table 3). In contrast, PAN_POS_ tended to show lower scores for the subscale “worries.”

Anxiety and depression symptoms did not differ from T0 to T1, neither for the whole PAN, nor for the subgroups PAN_NEG_ and PAN_POS_ (Tables 2, 3). For the whole PAN, the MCIDs for anxiety improved in 20%, worsened in 20% and remained stable in 60% of the patients. For depression, the MCIDs improved in 23%, worsened in 15% and remained stable in 63% of the patients.

**Figure 1:**
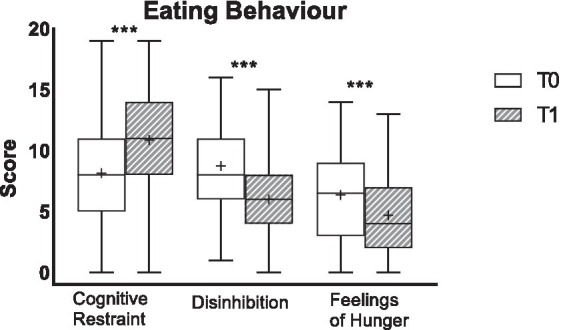
Eating behaviour of the total study population for pre- (T0) and post-intervention (T1). Scores for eating behaviour are presented. The data are shown as box-whiskers [median with upper and lower quartiles, whose difference describes the interquartile range (IQR) and minimum and maximum (=whiskers)]. Mean is depicted as “+.” Increases from T0 to T1 for cognitive restraint as well as decreases from T0 to T1 for disinhibition and feelings of hunger represent an improvement. ***Significant differences between T0 and T1 (FDR < 0.001).

For the whole PAN, all subscales of the eating behaviour questionnaire (TFEQ) improved significantly from baseline to the end of the intervention (Figure 1). More than half (58%) of the participants showed an improvement for “Cognitive restraint,” 16% worsened and 26% remained stable. “Disinhibition” improved in 55% of the patients, worsened in 6% and remained stable in 40%. Regarding “Feelings of hunger” from pre-to post-treatment, more than half of the patients (54%) improved, while 20% worsened and 26% remained similar. In line were the results for the two subgroups PAN_NEG_ and PAN_POS_: except for Feelings of Hunger for PAN_POS_ all three subscales improved from pre-to post-treatment. Eating behavior did not differ significantly between PAN_NEG_ and PAN_POS_ from pre-to post-treatment (FDR >0.1, Table 3).

#### 3.3.3 Participants benefit personally from the intervention

In general, the evaluation questionnaire at the end of the intervention showed a high satisfaction of the participants with the program. Almost all participants (90%) would recommend the intervention to family and friends. The results are shown in Supplementary material S3. Participants with worsening MCIDs did not show lower levels of satisfaction with the program than participants who showed improvement or had stable MCIDs. Detailed information is presented in the Supplementary material S4.

## 4 Discussion

This study investigated the impact of the COVID-19 pandemic on psychological parameters and eating behavior in people with predominantly grade III obesity who sought help from a multimodal conservative weight loss program during the pandemic. Contrary to our hypothesis (i), people with severe obesity who enrolled in a multimodal BWL program did neither show increased anxiety and depression symptoms nor higher perceived stress than prior to the pandemic. These findings are partially consistent with previous research. Studies recorded an increase in anxiety and depressive symptomatology and stress experience during the COVID-19 pandemic, in contrast to the present work (6, 15). However, this research related to the initial period of the pandemic, concomitant with the first lockdown. In the “Mannheim Corona Study,” which examined the impact of the COVID-19 pandemic on the German population over a sixteen-week period beginning in March 2020, symptoms of anxiety and depression were most pronounced shortly after the quarantine order. Over time, these symptoms decreased slightly. This may be due to a habituation effect (73) or, as trauma researchers have shown, to the fact that many people experience a phase of resilience or recovery after negative events (74). Studies that also focused on later time points in the pandemic like this (started in July 2020 up to April 2022) research showed similar results to the present (75, 76).

People living with obesity are highly affected by weight stigma and discrimination, which are associated with psychological distress (77): in the workplace, schools and colleges, in health care settings, and even in private settings (78–80). Strict contact restrictions and curfews were mandated to combat the COVID-19 pandemic, which may have led to a reduction in discrimination and stigmatization of people living with obesity (81, 82) and thus may have contributed to trending lower expressions of anxiety and depression symptomatology and stress experience. Furthermore, the certainty of experiencing food without an external evaluation may have led to these lower scores compared to before. Another possibility for the trend toward lower stress levels during the COVID-19 pandemic compared with before may be less experienced time stress since pandemic onset. The introduction and expansion of home office in various work sectors (83, 84) allowed for more flexible work schedules and decimated commutes and travel for many employees (85). In addition, many business trips were cancelled. Meetings with people in other places and countries were instead held online via videoconferencing, resulting in significantly reduced times spent in cars, buses, trains or planes (86).

Compared to PrePAN, PAN exhibited lower perceived hunger levels as well as lower disturbance of eating behavior at baseline. One reason for this may be a decreased opportunity for casual purchases, social eating situations, or restaurant visits. Some research suggests that as BMI increases, experienced feelings of hunger and the disturbance of eating behavior increases, while cognitive control decreases (8, 87, 88). Because many activities occurred within the home due to COVID-19 constraints and significantly less time was spent on the go (89), spontaneous purchases due to external factors such as the sight or smell of food may have decreased. Research shows that higher cognitive restraint scores are associated with lower BMI, lower energy intake, and lower appetite ratings (62) and leads to higher weight loss in weight reduction interventions (90). Nevertheless, it is also possible that although cognitive restraint may lead to lower BMI and greater weight loss during weight loss interventions, rigid cognitive restraint may also lead to the development of disordered eating behaviors (62, 91) and can therefore also have a negative impact.

As hypothesised (ii), BWL intervention during the COVID-19 pandemic led to stabilization and improvement in eating behaviour and physical and psychological parameters. At the end of the intervention, body weight reduction was observed in two-thirds of the subjects and body weight stabilization in 16% of the participants. There was an improvement or stabilization with respect to anxiety and depression symptoms in 86% and 80% of the patients, respectively. Consistent with our hypothesis, perceived stress (worry and demands) tended to decrease over the course of the intervention. However, PAN was significantly higher in stress experience both before and after the intervention than the sample of healthy adults studied by Fliege et al. (61).

Furthermore, PAN showed improvements in physical, but not psychological HRQoL at the end of the intervention. This is also documented in the existing literature: clinical BWL programs showed successful improvements in physical HRQoL (92–94). Improvements in HRQoL were shown independent of weight loss in various weight loss programs (9, 94). Interestingly, participants who showed a worsening of psychological parameters during treatment, rated the BWL program as positive as those with improvement or stabilization. This suggests that the participants’ benefit extends beyond the reduction of psychological symptoms.

Patients in medical and psychotherapeutic treatments during the pandemic were especially grateful that therapeutic treatment could take place despite the pandemic (95). This may have been the same for the patients in this study. The program may have prevented the development of even worse psychological outcomes in these patients.

PAN showed more beneficial eating behavior after the intervention when compared to baseline. Previous studies showed that eating behavior change positively during weight loss interventions, but that the disturbance of eating behavior and experienced feelings of hunger are not directly associated with body weight change (47, 96, 97). This is supported by the present results.

The presented study has strengths and limitations. A clear strength of the study is the low dropout rate of 8%, whereby the large sample provides meaningful results for further theoretical assumptions and practical implementations. Only validated questionnaires were used to assess participants’ anxiety and depression symptoms, stress experience, HRQoL, and eating behavior, which is another clear strength of the work. Missing values were replaced using the predictive mean matching method to calculate values for the ITT population. During the COVID-19 pandemic body weight was subjectively reported with data appearing realistic. One study showed that self-reported body weight is underestimated in people with obesity (49) whereas other studies report that body weight is also accurately reported in people with overweight and obesity (50), especially in those participating in a weight loss program (51). The data of this study could not be compared to a control group. Theoretically, a comparison with the PrePAN group would have been possible but we decided, that this comparison was not acceptable because two variables would have been mixed at the same time, being the life condition (pre-pandemic versus pandemic) and mode of delivery of the BWL intervention (face-to-face versus online). The differences between PrePAN and PAN regarding anxiety and depression symptomatology as well as stress experience disappeared after comparing age-, gender-, BMI-, and surgery-attitude-matched subjects between the two groups. The reason could be due to the only minor differences in the total sample, which were lost after matching the sample groups due to the reduced sample size.

## 5 Conclusion

People with severe obesity attending a virtual, video-based multimodal BWL intervention program during COVID-19 were similar burdened with regard to HRQoL and symptoms for anxiety and depression, stress, when compared to pre-pandemic times. A multidisciplinary conservative weight loss intervention in a virtual setting during the uncertain times of the pandemic helped to stabilize and improve the physiological and psychological burdens of people with severe obesity. Although, digitization due to the COVID-19 pandemic has rapidly advanced the progress of technology in healthcare, digital options in the field of obesity treatment should be further expanded and evaluated to maximize the effectiveness of these interventions.

## Data availability statement

The raw data supporting the conclusions of this article will be made available by the authors, without undue reservation.

## Ethics statement

The studies involving humans were approved by Ethics Committee of the University Hospital Tübingen, Germany. The studies were conducted in accordance with the local legislation and institutional requirements. The participants provided their written informed consent to participate in this study.

## Author contributions

JS: Formal analysis, Methodology, Visualization, Writing – original draft. KB: Formal analysis, Methodology, Writing – review & editing. SS: Methodology, Writing – review & editing. BK: Methodology, Writing – review & editing. RE: Methodology, Writing – review & editing. AS: Supervision, Writing – review & editing. AN: Resources, Writing – review & editing. SZ: Resources, Writing – review & editing. IM: Conceptualization, Formal analysis, Methodology, Resources, Supervision, Visualization, Writing – original draft.
