# Supplementary material for: Conventional weight loss therapy in morbid obesity during COVID-19 pandemic: degree of burdens at baseline and treatment efficacy
Source: Front Psychiatry. 2024 Jan 22;15:1330278. doi: 10.3389/fpsyt.2024.1330278 (PMC10839038; doi:10.3389/fpsyt.2024.1330278)
Supplement: Supplementary file 1 [file Table_1.docx]

**Supplementary Material 1:** Baseline characterization for the two sample groups matched by age, sex, initial BMI, and attitude towards bariatric surgery

|  | **prePAN_Match_** (n = 141)  mean (SD) [95 % KI] | | | **PAN_Match_** (n = 141)  mean (SD) [95 % CI] | | **Statistics for prePAN vs. PAN**  Mann-Whitney U Test/χ2 |
| --- | --- | --- | --- | --- | --- | --- |
| Age, years | 42.1 (12.2) [40.1–44.2] | | | 42.5 (12.8) [40.4-44.7] | | *U* = 9812.000, *p* = 0.851 |
| BMI  Range: min to max | 42.5 (5.3) [41.6–43.3]  27.6-63.3 | | | 42.6 (5.5) [41.7-43.5]  28.9-66.3 | | *U* = 9885.500, *p* = 0.936 |
| Weight  Range: min to max | 121.9 (19.5) [118.6–125.1]  77.8-177.0 | | | 122.7 (19.0) [119.6-125.9]  75.8-185.0 | | *U* = 9635.500, *p* = 0.656 |
|  | *N* (%) | | | *N* (%) | |  |
| Sex (female) | 112 (79) | | | 112 (79) | | χ2 (1, *N* = 258) = 0.026,  *p* = 0.872, φ = -0.010 |
| Nationality  German/foreigner | 115 (90) / 26 (10) | | | 116 (89) / 25 (11) | |  |
| Smoker | 24 (19) | | | 24 (18) | | χ2 (1, *N* = 260) = 0.056,  *p* = 0.813, φ = -0.015 |
| Bariatric surgery desire  No  Yes  Not clear | 86 (61)  52 (37)  3 (2) | | | 86 (61)  52 (37)  3 (2) | |  |
| Personal Status  Single  Married  Separated  Divorced  Widowed  Others | 35 (28)  69 (56)  2 (2)  13 (11)  2 (2)  3 (2) | | | 33 (25)  83 (62)  2 (2)  13 (10)  2 (2)  1 (1) | | χ2_FFH_ (5, *N* = 258) = 1.964,  *p* = 0.854, φ = 0.087 |
| Composition of household  Alone  With Partner  Alone with child(ren)  Partner and child(ren)  With parents  Others | 17 (14)  39 (32)  11 (9)  41 (33)  9 (7)  7 (6) | | | 19 (14)  39 (30)  10 (8)  48 (36)  12 (9)  4 (3) | | χ2 (6, *N* = 256) = 3.002,  *p* = 0.809, φ = 0.108 |
| Level of education  Sec. mod. school  Polytechnic  Sec. techn. school  High school  University  Others | 31 (25)  0 (0)  50 (40)  16 (13)  24 (19)  2 (2) | | | 38 (29)  0  50 (38)  31 (23)  12 (9)  2 (2) | | χ2 (6, *N* = 257) = 10.195,  *p* = 0.070, φ = 0.199 |
|  | **prePAN** |  | | **PAN** | | **Statistics for prePAN vs. PAN**  Mann Whitney U test |
|  | Mean (SD) [95% CI] | | Median [IQR] | Mean (SD) [95% CI] | Median [IQR] |  |
| HRQoL (SF-12)  MCS  PCS | 43.2 (11.7) [41.2-45.2]  36.5 (11.4) [34.5-38.4] | | 44.0 [35.1-53.3]  36.0 [28.6-44.7] | 44.1 (11.8) [42.1-46.1]  33.5 (10.7) [31.6-35.3] | 43.9 [35.1-55.3]  32.2 [24.7-42.5] | *U* =8667.000, *FDR* = 0.604  *U* = 7645.000, *FDR*= 0.113 |
| Anxiety (GAD-7) Score | 7.5 (4.8)  [6.7-8.4] | | 7 [4-11] | 6.7 (4.6)  [5.9-7.5] | 6 [3-9] | *U* = 8062.500, *FDR* = 0.294 |
| Depression (PHQ-9) Score | 8.6 (5.1)  [7.8-9.5] | | 8 [5-11] | 8.1 (5.0)  [7.3-9.0] | 7 [4-11] | *U* = 8384.000, *FDR* = 0.399 |
| Eating behavior (TFEQ) Scores  TFEQ-subscale: cognitive restraint  TFEQ-subscale: disinhibition  TFEQ-subscale: feelings of hunger | 8.4 (3.7) [7.7-9.0]  9.8 (3.6) [9.2-10.4]  7.4 (3.5) [6.8-8.0] | | 8 [6-11]  10 [8-13]  8 [5-10] | 8.1 (4.5) [7.3-8.8]  8.7 (3.6) [8.1-9.3]  6.3 (3.5) [5.7-6.9] | 7.5 [5-11]  8 [6-11]  6 [3-9] | *U* = 8350.000, *FDR* = 0.399  *U* = 7314.500, *FDR* = 0.065,  *r* = -0.17  *U* = 7425.000, *FDR* = 0.065,  *r* = -0.18 |
| Perceived Stress (PSQ20) Scores  Sum  Worries  Tension  Joy  Demands | 49.6 (20.9) [45.8-53.3]  46.0 (25.7) [41.4-50.6]  52.3 (23.8) [48.0-56.6]  47.9 (22.4) [43.9-51.9]  47.9 (25.7) [43.3-52.4] | | 52.5 [33.3-66.7]  46.6 [20-66.7]  53.3 [33.3-73.3]  46.7 [31.7-73.3]  46.7 [31.7-66.7] | 46.6 (20.2) [43.1-50.0]  43.1 (26.3) [38.6-47.6]  49.9 (24.3) [45.8-54.1]  49.0 (20.8) [45.5-52.6]  42.3 (23.0) [38.4-46.2] | 43.3 [33.3-63.3]  40 [26.7-61.7]  46.7 [33.3-66.7]  46.7 [33.3-60]  40 [26.7-55] | *U =* 7401.000, *FDR* = 0.383  *U =* 7566.500, *FDR* = 0.404  *U =* 7654.500, *FDR* = 0.454  *U* = 7883.000, *FDR* = 0.621  *U =* 7044.500, *FDR* = 0.166 |
| **Abbreviations**: BMI, Body Mass Index; CI, Confidence Interval; FDR, False Discovery Rate; GAD, Generalized Anxiety Disorder Scale; IQR, Interquartile range; HRQoL, Health-related Quality of Life; MCS, Mental Component Score; n, sample size; PCS, Physical Component Score; PHQ, Patient Health Questionnaire; prePAN, Patients participating in the intervention prior to the COVID-19 pandemic; PAN, patients participating in the intervention since the COVID-19 pandemic; PSQ, Perceived Stress Questionnaire; SD, Standard derivation; SF, Short Form Health survey; TFEQ, Three factor Eating Questionnaire; T0, In the beginning of the Intervention; T1, At the end of the intervention. **Statistics**: U = Mann-Whitney U test; χ2 = χ2 test; φ = effect size (for χ2 test); p < .001 is considered statistically significant; FDR < .05 is considered statistically significant, FDR .05-.1 is considered as a trend, r = effect size (for Mann-Whitney U test). | | | | | | |
