# Supplementary material for: Conventional weight loss therapy in morbid obesity during COVID-19 pandemic: degree of burdens at baseline and treatment efficacy
Source: Front Psychiatry. 2024 Jan 22;15:1330278. doi: 10.3389/fpsyt.2024.1330278 (PMC10839038; doi:10.3389/fpsyt.2024.1330278)
Supplement: Supplementary file 2 [file Table_2.docx]

|  | ***Total*** | ***Non- Responder: ≤ 0 %*Body weight loss** | ***Responder:  0-4.9 %*Body weight loss** | ***Responder: 5-9.9 %*Body weight loss** | ***Responder:  ≥ 10 %*Body weight loss** | ***Non-Completer*** |
| --- | --- | --- | --- | --- | --- | --- |
| **PAN** |  |  |  |  |  |  |
| **N_PP_ (%)** | 133 (100) | 39 (29) | 69 (52) | 22 (17) | 3 (2) | 13 (-) |
| **∆Body weight_PP_ [kg]** | -1.9 | +4.0 | -2.4 | -8.5 | -16.8 | n.d. |
| **Mean BMI_PP_***  Overweight n (%)  Class I obesity n (%)  Class II obesity n (%)  Class III obesity n (%) | 42.1  1 (1)  8 (6)  43 (32)  81 (61) | 43.7  0 (0)  2 (5)  9 (23)  28 (72) | 42.1  0 (0)  3 (4)  24 (35)  42 (61) | 40.2  0 (0)  2 (9)  10 (46)  10 (46) | 33.2  1 (33)  1 (33)  0 (0)  1 (33) | n.d.  n.d.  n.d.  n.d.  n.d. |
| **N_ITT_ (%)** | 146 (100) | 52 (36) | 69 (47) | 22 (15) | 3 (2) | 13 (-) |
| **∆Body weight_ITT_ [kg]** | -1.9 | +3.1 | -2.8 | -8.5 | -16.8 | n.d. |
| **PAN_NEG_** |  |  |  |  |  |  |
| **N_PP_ (%)** | 80 (100) | 19 (24) | 41 (51) | 18 (23) | 2 (3) | 10 (-) |
| **∆Body weight_PP_ [kg]** | -2.8 | +2.7 | -2.3 | -8.8 | -12.5 | n.d. |
| **Mean BMI_PP_***  Overweight n (%)  Class I obesity n (%)  Class II obesity n (%)  Class III obesity n (%) | 40.8  1 (1)  7 (9)  34 (43)  38 (48) | 41.9  0 (0)  2 (11)  6 (32)  11 (58) | 41.0  0 (0)  3 (7)  18 (44)  20 (49) | 40.5  0 (0)  1 (6)  10 (56)  7 (39) | 29.4  1 (50)  1 (50)  0 (0)  0 (0) | n.d.  n.d.  n.d.  n.d.  n.d. |
| **N_ITT_ (%)** | 90 (100) | 26 (29) | 44 (49) | 18 (20) | 2 (2) | 10 (-) |
| **∆Body weight_ITT_ [kg]** | -2.7 | +2.2 | -2.7 | -8.8 | -12.5 | n.d. |
| **PAN_POS_** |  |  |  |  |  |  |
| **N_PP_ (%)** | 50 (100) | 20 (40) | 26 (52) | 3 (6) | 1 (2) | 0 (-) |
| **∆Body weight_PP_ [kg]** | -0.2 | +5.2 | -2.4 | -7.6 | -16.8 | n.d. |
| **Mean BMI_PP_***  Class I obesity n (%)  Class II obesity n (%)  Class III obesity n (%) | 44.1  1 (2)  8 (16)  41 (80) | 45.4  0 (0)  3 (15)  17 (85) | 43.8  0 (0)  5 (19)  21 (81) | 38.2  1 (33)  0 (0)  2 (67) | 40.8  0 (0)  0 (0)  1 (100) | n.d.  n.d.  n.d.  n.d. |
| **N_ITT_ (%)** | 53 (100) | 26 (49) | 23 (43) | 3 (6) | 1 (2) | 0 (-) |
| **∆Body weight_PP_ [kg]** | -0.2 | +4.0 | -2.9 | -7.6 | -25.3 | n.d. |

**Supplementary Material 2**. Body weight change

**Abbreviations**: ∆Body weight, change in body weight; BMI, Body Mass Index; Class I obesity, BMI of 30-34.9 kg/m^2^; Class II obesity, BMI of 35-39.9 kg/m^2^; Class III obesity, BMI of ≥ 40 kg/m^2^; ITT, Intention to treat population; N, sample size; PAN, Patients participating in the intervention during the COVID-19 pandemic; PAN_NEG_, participants of the PAN group who had a negative attitude towards bariatric surgery; n.d., no data; Non-Completer, <80% participation in the program; Non-Responder, no body weight loss or body weight gain during intervention; PP, Per Protocol Population; PAN_POS_, participants of the PAN group who had a positive attitude towards bariatric surgery; Responder, body weight loss (<0kg) during six-month intervention; Overweight, BMI of 25-29.9 kg/m^2^;*data from Per-Protocol-population.
