# Supplementary material for: Conventional weight loss therapy in morbid obesity during COVID-19 pandemic: degree of burdens at baseline and treatment efficacy
Source: Front Psychiatry. 2024 Jan 22;15:1330278. doi: 10.3389/fpsyt.2024.1330278 (PMC10839038; doi:10.3389/fpsyt.2024.1330278)
Supplement: Supplementary file 3 [file Table_3.docx]

**Supplementary Material 3:** Evaluation of the total group. *N* = 89

Supplemental Material 3 shows the results of the evaluation questionnaire regarding participants‘ personal benefits from the intervention on a 5-point Likert scale.
