# Supplementary material for: Conventional weight loss therapy in morbid obesity during COVID-19 pandemic: degree of burdens at baseline and treatment efficacy
Source: Front Psychiatry. 2024 Jan 22;15:1330278. doi: 10.3389/fpsyt.2024.1330278 (PMC10839038; doi:10.3389/fpsyt.2024.1330278)
Supplement: Supplementary file 4 [file Table_4.docx]

**Supplementary Material 4:** Comparison of the evaluation of the subgroups with MCIDs worsened or MCIDs improved or remained stable

|  | **GAD-7** | | | **PHQ-9** | | | **SF-12 mental** | | | **SF-12 physical** | | |
| --- | --- | --- | --- | --- | --- | --- | --- | --- | --- | --- | --- | --- |
|  | **MCID worsened**  n = 27  **mean (SD)**  **[95% CI]**  **median**  **[IQR]** | **MCID improved**  **or remained stable**  n = 111  **mean (SD)**  **[90% CI]**  **median**  **[IQR]** | **Statistics**  Mann-Whitney-U-Test | **MCID worsened**  n = 20  **mean (SD)**  **[95% CI]**  **median**  **[IQR]** | **MCID improved**  **or remained stable**  n = 118  **mean (SD)**  **[90% CI]**  **median**  **[IQR]** | **Statistics**  Mann-Whitney-U-Test | **MCID worsened**  n = 50  **mean (SD)**  **[95% CI]**  **median**  **[IQR]** | **MCID improved**  **or remained stable**  n = 88  **mean (SD)**  **[90% CI]**  **median**  **[IQR]** | **Statistics**  Mann-Whitney-U-Test | **MCID worsened**  n = 47  **mean (SD)**  **[95% CI]**  **median**  **[IQR]** | **MCID improved**  **or remained stable**  n = 91  **mean (SD)**  **[90% CI]**  **median**  **[IQR]** | **Statistics**  Mann-Whitney-U-Test |
| Personal benefit | 3.6 (1.0) [3.1-4.2]  3.0 [3.0-4.0] | 3.8 (1.0)  [3.5-4.0]  4.0 [3.0-4.0] | U = 454.000  *p =* .727 | 3.8 (1.0)  [3.2-4.4]  4.0 [3.0-4.8] | 3.7 (1.0)  [3.4-4.0]  4.0  [3.0-4.0] | U = 347.500  *p =* .583 | 3.8 (0.9) [3.3-4.2]  4.0 [3.0-4.0] | 3.7 (1.0)  [3.4-4.0]  4.0 [3.0-4.0] | U = 534.000  *p =* .924 | 3.7 (1.2) [3.2-4.1]  4.0 [3.0-4.0] | 3.8 (1.0) [3.5-4.0]  4.0 [3.0-4.0] | U = 566.000  *p =* .332 |
| Importance and usefulness of the nutrition session | 4.1 (0.7) [3.7-4.5]  4.0 [4.0-4.8] | 4.1 (0.8)  [3.9-4.3]  4.0 [4.0-5.0] | U = 459.500  *p =* .778 | 4.2 (0.7)  [3.7-4.6]  4.0 [4.0-5.0] | 4.1 (0.8)  [3.8-4.3]  4.0 [4.0-5.0] | U = 363.000  *p =* .746 | 4.1 (0.6) [3.7-4.4]  4.0 [4.0-4.0] | 4.1 (0.8)  [3.9-4.4]  4.0 [4.0-5.0] | U = 483.500  *p =* .452 | 4.1 (0.8) [3.8-4.5]  4.0 [4.0-5.0] | 4.1 (0.8) [3.9-4.3]  4.0 [4.0-5.0] | U = 633.000  *p =* .957 |
| Importance and usefulness of stress management | 4.0 (1.0)  [3.5-4.5]  4.0 [3.3-5.0] | 4.1 (0.8)  [3.9-4.3]  4.0 [3.3-5.0] | U = 474.500  *p =* .941 | 4.2 (1.0)  [3.5-4.8]  4.0 [3.3-5.0] | 4.1 (0.8)  [3.6-4.3]  4.0 [3.8-5.0] | U = 339.500  *p =* .500 | 4.1 (0.9) [3.6-4.5]  4.0 [3.0-5.0] | 4.1 (0.8) [3.9-4.3]  4.0 [3.5-5.0] | U = 540.500  *p =* .990 | 4.1 (0.8) [3.7-4.4]  4.0 [3.0-5.0] | 4.1 (0.9)  [3.8-4.3]  4.0 [4.0-5.0] | U = 580.500  *p =* .418 |
| Importance and usefulness of the advice on exercise | 3.9 (1.0)  [3.3-4.4]  4.0 [3.0-5.0] | 3.9 (1.0)  [3.7-4.2]  4.0 [3.0-5.0] | U = 471.000  *p =* .904 | 4.1 (1.0)  [3.5-4.7]  4.0 [3.3-5.0] | 3.9 (1.0)  [3.6-4.2]  4.0 [3.0-5.0] | U = 333.000  *p =* .445 | 4.0 (1.0)  [3.5-4.5]  4.0 [3.0-5.0] | 3.9 (1.0)  [3.6-4.2]  4.0 [3.0-5.0] | U = 546.000  *p =* .862 | 4.0 (1.0)  [3.6-4.4]  4.0 [3.0-5.0] | 3.9 (1.0) [3.6-4.2]  4.0 [3.0-5.0] | U = 617.500  *p =* .708 |
| Adequacy of the level of exercising | 3.7 (1.2) [3.0-4.3]  4.0 [3.0-4.8] | 3.8 (1.0)  [3.6-4.1]  4.0 [3.0-4.3] | U = 480.000  *p =* .835 | 4.0 (1.3)  [3.1-4.9]  4.5 [3.3-5.0] | 3.8 (1.0)  [3.5-4.0]  4.0 [3.0-4.0] | U = 300.500  *p =* .164 | 3.9 (1.1) [3.4-4.5]  4.0 [3.3-5.0] | 3.7 (1.0) [3.5-4.0]  4.0 [3.0-4.0] | U = 478.000  *p =* .219 | 3.7 (1.0)  [3.3-4.1]  4.0 [3.0-4.0] | 3.8 (1.1) [3.5-4.2]  4.0 [3.0-5.0] | U = 644.000  *p =* .623 |
| Comprehensibility of the instructions of exercising | 4.4 (0.8)  [3.9-4.9]  5.0 [4.0-5.0] | 4.6 (0.5)  [4.4-4.7]  5.0 [4.0-5.0] | U = 432.000  *p =* .770 | 4.6 (0.7) [4.2-5.0]  5.0 [4.0-5.0] | 4.4 (0.6)  [4.4-4.7]  5.0 [4.0-5.0] | U = 365.500  *p =* .690 | 4.5 (0.7) [4.2-4.9]  5.0 [4.0-5.0] | 4.6 (0.6) [4.4-4.7]  5.0 [4.0-5.0] | U = 546.500  *p =* .752 | 4.8 (0.4) [4.7-5.0]  5.0 [5.0-5.0] | 4.4 (0.7) [4.2-4.6]  4.0 [4.0-5.0] | U = 402.500  ***p* < .001** |
| Motivation to implement more exercise in daily life | 3.7 (0.8)  [3.3-4.2]  4.0 [3.0-4.0] | 3.6 (0.9)  [3.4-3.9]  4.0 [3.0-4.0] | U = 474.000  *p =* .770 | 3.9 (0.9) [3.3-4.5]  4.0 [3.3-4.8] | 3.6 (0.9)  [3.4-3.8]  4.0 [3.0-4.0] | U = 324.000  *p =* .284 | 3.7 (0.8) [3.3-4.1]  4.0 [3.0-4.0] | 3.7 (1.0)  [3.4-4.0]  4.0 [3.0-4.0] | U = 550.000  *p =* .712 | 3.8 (0.9) [3.4-4.2]  4.0 [3.0-4.0] | 3.6 (0.9)  [3.3-3.9]  4.0 [3.0-4.0] | U = 644.500  *p =* .619 |
| Feeling prepared for the time after the program | 3.4 (0.7)  [3.0-3.8]  3.0 [3.0-4.0] | 3.7 (0.9)  [3.5-3.9]  4.0 [3.0-4.0] | U = 413.000  *p =* .269 | 3.5 (1.1)  [2.8-4.2]  4.0 [2.3-4.0] | 3.6 (0.7) [3.4-3.8]  4.0 [3.0-4.0] | U = 381.000  *p =* .823 | 3.6 (0.9) [3.2-4.0]  4.0 [3.0-4.0] | 3.6 (0.8)  [3.4-3.9]  4.0 [3.0-4.0] | U = 520.000  *p =* .460 | 3.7 (0.8)  [3.3-4.1]  4.0 [3.0-4.0] | 3.6 (0.8) [3.3-3.8]  4.0 [3.0-4.0] | U = 651.500  *p =* .676 |
| Optimism to maintain or further reduce the weight | 3.4 (0.6)  [3.1-3.8]  3.0 [3.0-4.0] | 3.4 (1.0)  [3.1-3.7]  3.0 [3.0-4.0] | U = 440.000  *p =* .740 | 3.3 (1.0)  [2.7-4.0]  3.0 [2.3-4.0] | 3.4 (0.9) [3.2-3.7]  3.0 [3.0-4.0] | U = 352.000  *p =* .757 | 3.4 (0.9) [3.0-3.9]  3.0 [3.0-4.0] | 3.4 (1.0) [3.1-3.7]  3.0 [3.0-4.0] | U = 490.000  *p =* .852 | 3.3 (1.2) [2.8-3.7]  3.0 [2.0-4.0] | 3.5 (0.9) [3.2-3.8]  4.0 [3.0-4.0] | U = 492.500  *p =* .148 |

**Abbreviations:** CI, Confidence Interval; GAD-7, Generalized Anxiety Disorder Questionnaire; IQR, Interquartile Range; MCID, minimal clinically important difference; n, sample size; PHQ-9, Patient Health Questionnaire; SD, Standard deviation; SF-12, Quality of Life Questionnaire. Statistics: U = Mann-Whitney-U test, p < .001 is considered as statistically significant. Significant changes are marked in bold.
